# Idiopathic Spinal Cord Herniation With Severe Adhesions Treated Using the Hammock Method: A Case Report

**DOI:** 10.7759/cureus.98678

**Published:** 2025-12-08

**Authors:** Koki Aizawa, Masaki Tatsumura, Shun Okuwaki, Katsuya Nagashima, Yosuke Takeuchi, Toru Funayama

**Affiliations:** 1 Division of Spine Center, Tsukuba University Hospital Mito Clinical Education and Training Center/Mito Kyodo General Hospital, Mito, JPN; 2 Department of Orthopedic Surgery, University of Tsukuba, Tsukuba, JPN

**Keywords:** brown-séquard syndrome, dural patch closure, expanded polytetrafluoroethylene (eptfe) sheet, hammock method, idiopathic spinal herniation, kinking type, serious adhesions, sling technique

## Abstract

Idiopathic spinal cord herniation has traditionally been considered a relatively rare disease. It presents as a slowly progressive myelopathy and necessitates surgical treatment. Herein, we describe a case of idiopathic spinal cord herniation treated with the hammock method.

The patient was a 63-year-old male who became aware of numbness in his left toe twelve months prior to his initial visit. At that time, he had difficulty standing on his right leg. Sensation for pain and temperature was decreased in the left half of his body below the navel. Bilateral lower extremity tendon reflexes were hyperactive, and the bilateral Babinski reflex was positive. He also reported a weak urine stream. The Japanese Orthopedic Association (JOA) score for the thoracic spine was 5 out of 11. Magnetic resonance imaging (MRI) revealed a dural defect at the T4 level, with spinal cord extramedullary prolapse and anterior deviation. Computed tomography (CT) myelography revealed a dural defect in the same area.

The patient presented with Frankel classification grade C paraplegia and a slowly progressive Brown-Séquard syndrome. He was diagnosed with idiopathic spinal cord herniation. We performed dural patch closure using the hammock method. Transcranial electrically stimulated muscle evoked potentials and somatosensory evoked potentials were used for monitoring during surgery.

After a laminectomy from T3 to T6, a mid-longitudinal incision was made through the dura and arachnoid to expose the spinal cord. The denticulate ligaments were dissected bilaterally, and the nerve roots were preserved. Due to severe adhesions, we expanded the cephalic defect. Next, we passed a non-powdered nitrile sheet anteriorly under the spinal cord, suspending it. The adhesions around the defect and the spinal cord were completely removed, and the spinal cord emerged. An expanded polytetrafluoroethylene (ePTFE) sheet was then inserted to replace the nitrile sheet and sutured to the dura mater as a full-perimeter patch to cover the defect. Postoperatively, there was no progression of paralysis, the anterior deviation of the spinal cord disappeared, and no recurrence was observed two years after surgery. Although he still needed a walking stick, his walking speed improved, and his sensory impairment improved. Final JOA score for the thoracic spine was 8 out of 11.

## Introduction

Idiopathic spinal cord herniation has traditionally been considered a relatively rare disease. In recent years, the number of reported cases has increased due to improvements in diagnostic imaging techniques. Idiopathic spinal cord herniation presents with slowly progressive myelopathy that does not resolve spontaneously, making it an indication. The following surgical methods have been reported: (1) hiatal enlargement, (2) dural suture, and (3) dural patch. The dural patch method requires manipulation in the anterior part of the spinal cord, which limits the working space and makes the procedure complicated [1]. The advantage of the hammock method is that the spinal cord is gently lifted from behind, allowing for manipulation from the dorsal side, which provides a wide working space. This eliminates the complexity of the procedure and allows for safe and reliable manipulation [2]. In this report, we describe a case of idiopathic spinal cord herniation treated with the hammock method.

## Case presentation

**Figure 1:**
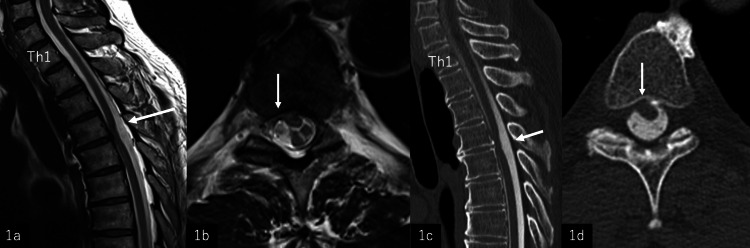
MRI and CT myelogram at initial visit 1a: T2-weighted midsagittal MRI at initial visit. Anterior deviation of the spinal cord is observed from the T4 to T5 level (arrow). 1b: T2-weighted axial MRI at the T4-T5 level at initial visit. The spinal cord is displaced anteriorly, and the anterior subarachnoid space has disappeared (arrow). 1c: Sagittal CT myelogram. The spinal cord is displaced anteriorly at the T4-T5 level and adheres to the arachnoid and dura mater (arrow). 1d: Axial CT myelogram at the T4-T5 level. The spinal cord is displaced right-anteriorly and adheres to the arachnoid and dura mater on the right side (arrow).

The patient was a 63-year-old male who presented with numbness in his left toe 12 months before the initial visit. At the time of the initial visit, the patient had difficulty standing on the right leg. Manual muscle testing showed that the right iliopsoas muscle was weakened to a score of 3 and the right quadriceps muscle to a score of 4. Sensation of pain and temperature was decreased in the left half of the body below the navel. Bilateral lower extremity tendon reflexes were hyperactive, and the Babinski reflexes on both sides were positive. He was aware of a weak urine stream. The Japanese Orthopedic Association (JOA) score for the thoracic spine was 5 out of 11. MRI revealed a dural defect at the 4th thoracic level, spinal cord extramedullary prolapse, and anterior deviation (Figure 1a, 1b). CT myelography revealed a dural defect in the same area (Figure 1c, 1d).

Sagittal images of MR and CT myelography showed a kinking type, and axial images of MR and CT myelography showed the defect located in the central region.

The patient presented with Frankel classification grade C paraplegia and Brown-Séquard syndrome with slowly progressive worsening. It was diagnosed as an idiopathic spinal cord herniation. We performed a dural patch closure with the hammock method. Transcranial electrically stimulated muscle evoked potentials and somatosensory evoked potentials were used during surgery.

**Figure 2:**
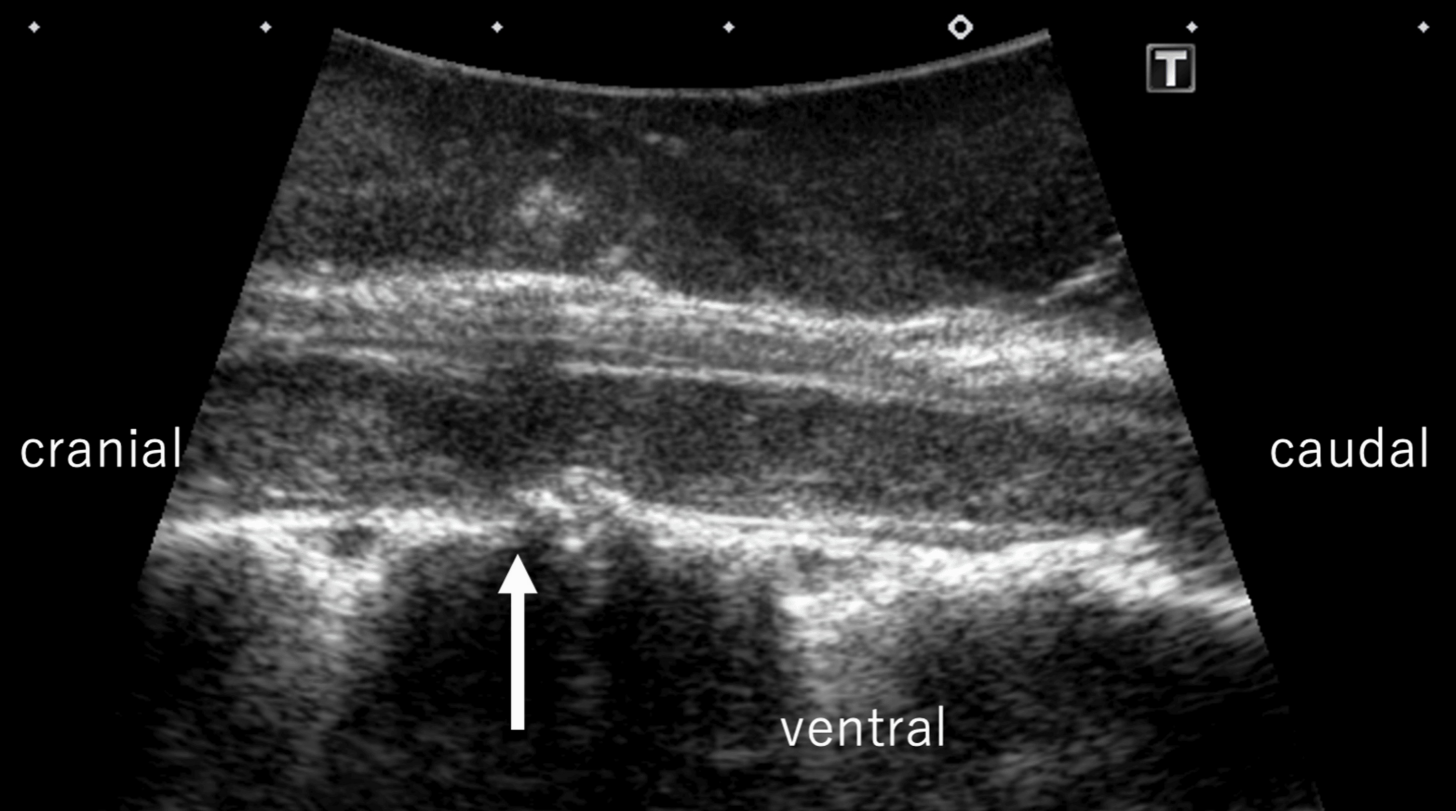
Ultrasound image taken during surgery Intraoperative ultrasound image. The spinal cord is shifted anteriorly, with expansion of the posterior subarachnoid space and disappearance of the anterior subarachnoid space (arrow).

After a laminectomy from the 3rd thoracic to the 6th thoracic laminar, we used intraoperative ultrasonography to confirm the point of anterior deviation of the spinal cord (Figure 2).

**Figure 3:**
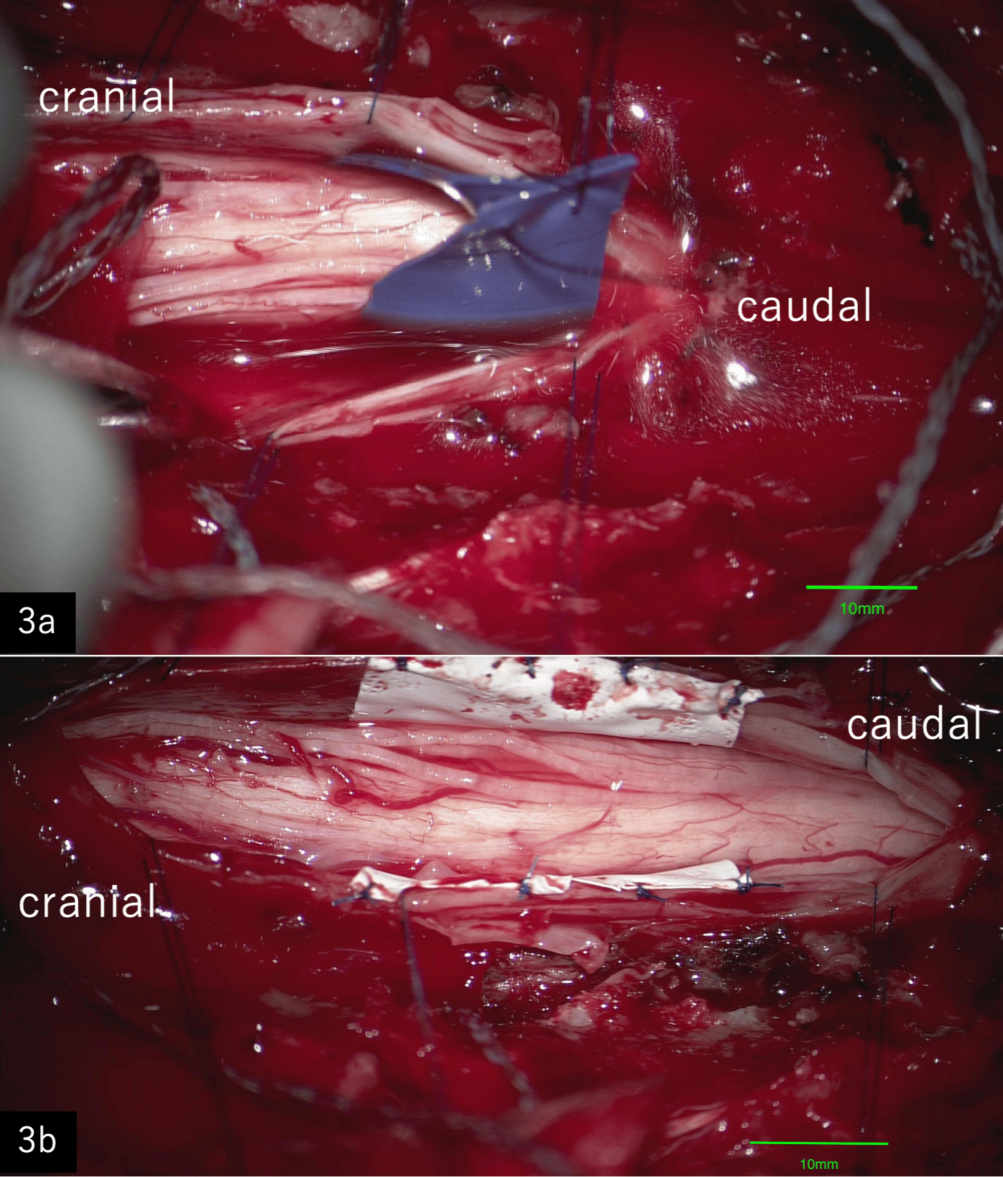
Intraoperative microscopic image 3a: Intraoperative microscopic image shows the spinal cord being gently lifted after passing a nitrile sheet ventrally. 3b: Intraoperative microscopic image. An expanded polytetrafluoroethylene (ePTFE) sheet has been inserted to cover the hiatus and prevent contact with the spinal cord.

A mid-longitudinal incision was made through the dura and arachnoid to expose the spinal cord. The denticulate ligaments were bilaterally dissected, and the nerve roots were preserved. The caudal side of the defect was difficult to expand due to severe adhesions, so we expanded the cephalic defect. Next, we passed a piece of non-powdered nitrile sheet anteriorly under the spinal cord, and suspended the spinal cord (Figure 3a). The adhesions around the defect and the spinal cord were completely removed, and the spinal cord emerged. Expanded polytetrafluoroethylene (ePTFE) sheet was inserted as an exchange for the nitrile sheet and sutured to the dura mater as a full-perimeter patch to cover the defect (Figure 3b).

**Figure 4:**
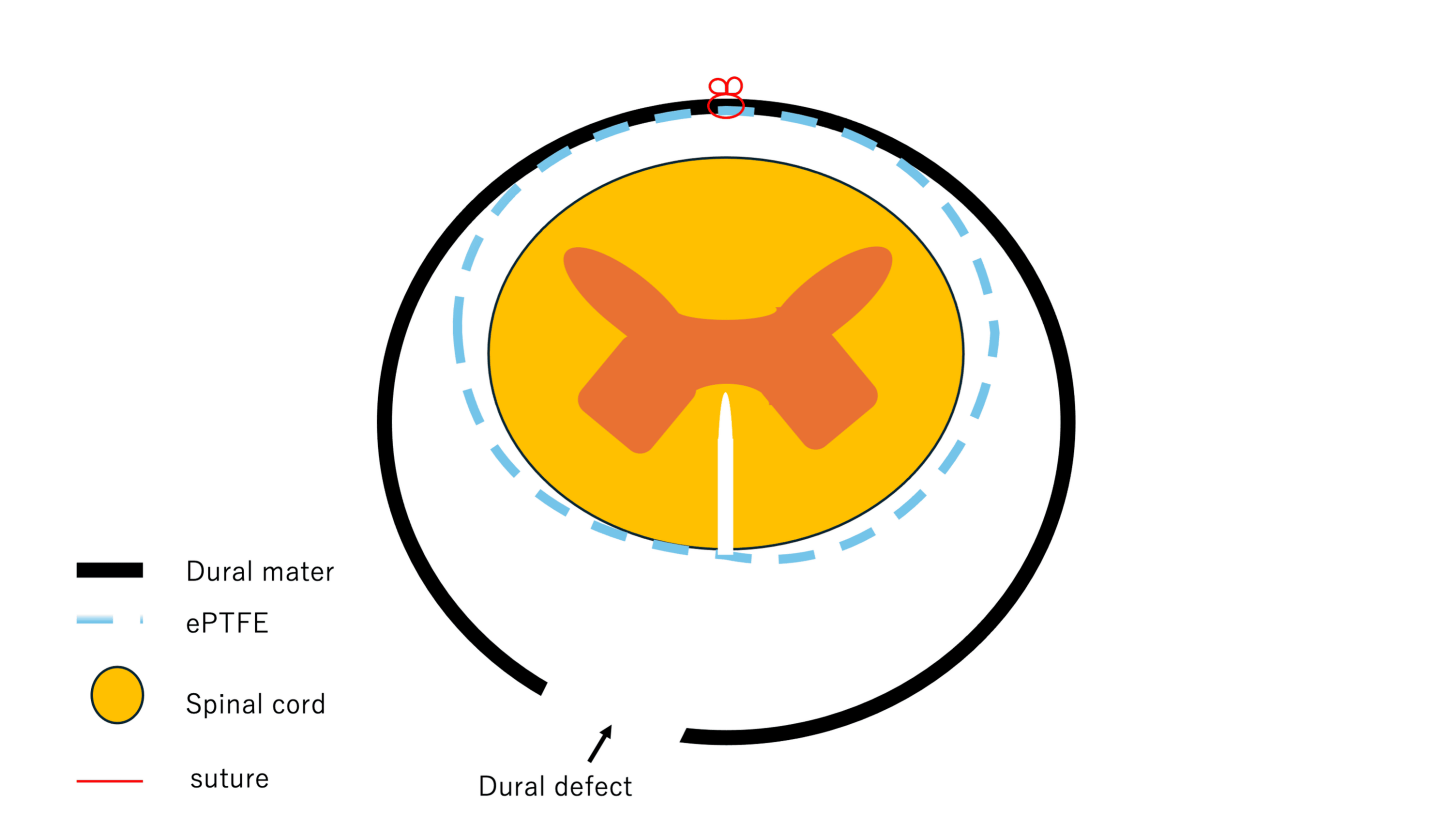
Schematic diagram of the hammock technique A schematic diagram of the hammock technique showing the relationship between the dura mater, dural defect, expanded polytetrafluoroethylene (ePTFE), and spinal cord.

The ePTFE and dura were sutured with 6-0 polypropylene (Figure 4).

**Figure 5:**
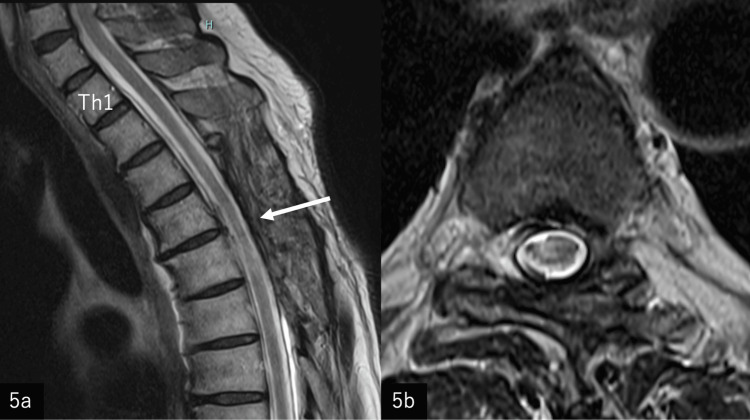
Post-operative MRI 5a: Postoperative T2-weighted midsagittal MRI two years after surgery. The anterior deviation of the spinal cord has improved, and the spinal cord is now centrally located within the dural canal (arrow). 5b: Postoperative T2-weighted axial MRI at the T4-T5 level. The anterior deviation of the spinal cord has improved, with the spinal cord now centrally located within the dural canal. Signal intensity changes are observed in some areas of the spinal cord.

Postoperatively, there was no progression of paralysis, the anterior deviation of the spinal cord disappeared (Figure 5a, 5b), and no recurrence was observed two years after surgery. Although he still needed a walking stick, his walking speed improved, and his sensory impairment improved. Final JOA score for the thoracic spine was 8 out of 11.

## Discussion

Idiopathic spinal cord herniation causes spinal cord symptoms due to a defect of the dura mater and adhesion of the spinal cord around the defect. In a meta-analysis of the previous 129 cases, the mean age was 51 (21-78) years, 36% were men, and 64% were women [3]. Slow progression of spinal symptoms was observed, and the average time to diagnosis was 17 months [4]. Idiopathic spinal cord herniation is most common in the thoracic spine. It is reported that Brown-Sequard syndrome is more common [3]. Imagama et al reported that findings on sagittal MRI and CT myelography were classified into three types: a kinking type, a discontinuous type, and a protrusion type; the patients with protrusion type herniation had a good postoperative recovery [1]. They also reported that findings on axial MR imaging and CT myelography axial images, the location of the defect was classified as either central or lateral, and the patients with a central defect had significant severe preoperative lower-extremity paralysis and a significantly poor postoperative recovery [1]. The present case was classified as a kinking type, and the defect location was central.

Surgical techniques for idiopathic spinal cord herniation reportedly include defect enlargement, dural suture, and dural patch closure [5]. The defect enlargement technique is safe and simple, but there is a possibility of re-excavation [5]. Dural suture can directly close the defect, but intraoperative manipulation may cause spinal cord injury [3]. Dural patch closure is a method of closing the defect using a patch and can reliably prevent re-excavation. On the other hand, the ventral side between the dura and the spinal cord has a smaller working space, making the procedure more complicated [6]. We chose the dural patch technique, which has a low recurrence rate. The hammock method, which is a type of dural patch technique, is used to ensure sufficient anterior space due to the severe adhesions at the dural defect. The hammock method [2] or the sling technique [7] provides sufficient working space by suspending the spinal cord and the ventral side of the dura. Therefore, we were able to perform safe adhesion removal. Spinal cord adhesion dissection is difficult without lifting, but direct grasping of the spinal cord poses the risk of spinal cord injury.

In this case, the hammock method was successfully applied for the treatment of idiopathic spinal cord herniation. A noteworthy strength of this case is that the technique allowed for sufficient surgical manipulation on the ventral side of the spinal cord while minimizing direct stress on the spinal cord itself, thereby ensuring both safety and effectiveness during the repair procedure.

There is also a risk of adhesion between the ePTFE and the spinal cord. In particular, there is a report of the formation of intradural cysts due to adhesion between the dura mater and ePTFE by using the hammock method [8]. In addition, in the kyphotic portion, the spinal cord is deviated anteriorly and in contact with the dura mater, which may pose a risk of spinal cord adhesions. There is also a report that performing the hammock method in a high position with severe local kyphosis resulted in spinal stenosis [7]. In our case, no abnormalities were observed after two years.

## Conclusions

In the present case, adhesions around the defect were severe, and the hammock method made it possible to lift the spinal cord and to detach the severe adhesions around the defect. In addition, a dural patch covered the defect to prevent re-evacuation. The hammock method provides sufficient working space by suspending the spinal cord, and the ventral side of the dura can be easily manipulated. The hammock method allows for safe and reliable operation and is considered to be a useful method.
